# Development of Omni InDel and supporting database for maize

**DOI:** 10.3389/fpls.2023.1216505

**Published:** 2023-06-28

**Authors:** Zhihao Liu, Yikun Zhao, Yunlong Zhang, Liwen Xu, Ling Zhou, Weiguang Yang, Han Zhao, Jiuran Zhao, Fengge Wang

**Affiliations:** ^1^ Key Laboratory of Crop DNA Fingerprinting Innovation and Utilization (Co-construction by Ministry and Province), Ministry of Agriculture and Rural Affairs, Beijing Academy of Agricultural and Forest Sciences (BAAFS), Beijing, China; ^2^ College of Agriculture, Jilin Agricultural University, Changchun, China; ^3^ Provincial Key Laboratory of Agrobiology, Institute of Crop Germplasm and Biotechnology, Jiangsu Academy of Agricultural Sciences, Nanjing, Jiangsu, China

**Keywords:** Omni InDel, maize, InDel, crops, database

## Abstract

Insertions–deletions (InDels) are the second most abundant molecular marker in the genome and have been widely used in molecular biology research along with simple sequence repeats (SSR) and single-nucleotide polymorphisms (SNP). However, InDel variant mining and marker development usually focuses on a single type of dimorphic InDel, which does not reflect the overall InDel diversity across the genome. Here, we developed Omni InDels for maize, soybean, and rice based on sequencing data and genome assembly that included InDel variants with base lengths from 1 bp to several Mb, and we conducted a detailed classification of Omni InDels. Moreover, we screened a set of InDels that are easily detected and typed (Perfect InDels) from the Omni InDels, verified the site authenticity using 3,587 germplasm resources from 11 groups, and analyzed the germplasm resources. Furthermore, we developed a Multi-InDel set based on the Omni InDels; each Multi-InDel contains multiple InDels, which greatly increases site polymorphism, they can be detected in multiple platforms such as fluorescent capillary electrophoresis and sequencing. Finally, we developed an online database website to make Omni InDels easy to use and share and developed a visual browsing function called “Variant viewer” for all Omni InDel sites to better display the variant distribution.

## Introduction

Insertions–deletions (InDels) rank second only to single-nucleotide polymorphisms (SNPs) as the most prevalent form of genetic variation in plants. Increasing numbers of studies have shown that InDels, which have the advantages of high polymorphism, co-dominance, high density, high reliability, and ease of genotyping, are the main source of plant genetic structural variation and are widely distributed in plant genomes (Das et al., 2015; Jain et al., 2019; Sun et al., 2019; Cui et al., 2021; Loewenthal et al., 2021; Pan et al., 2022). In practical applications, InDels are also favored by breeders compared to SNPs and simple sequence repeats (SSRs) because InDel detection merely necessitates straightforward techniques such as gel-based size separation or polyacrylamide gel electrophoresis, common procedures in genetics and breeding laboratories. (Păcurar et al., 2012; Liu et al., 2013; Wu et al., 2013; Moghaddam et al., 2014; Yang et al., 2014; Li et al., 2015; Zhou et al., 2015; Feng et al., 2020; Pan et al., 2021). Moreover, InDels are readily compatible with multi-platform detection techniques, including electrophoresis, chip arrays, and KASP tools that have been widely developed and applied for species diagnosis, marker-assisted selection breeding, evolutionary studies, genetic linkage map construction, and ancestral sequence reconstruction (Ashkenazy et al., 2012; Liu et al., 2013; Yamaki et al., 2013; Li et al., 2015; Song et al., 2015; Vialle et al., 2018; Jain et al., 2019; Pan et al., 2022; Seo et al., 2022). In addition, InDels are an important aspect of molecular evolutionary research because these variations shape genes and genomes, and some studies have shown their effects on proteins, including potential functional gains/losses in organisms (Lovett, 2004; Cartwright, 2009; Tian et al., 2008; Terakami et al., 2012; Rockah-Shmuel et al., 2013; Lin et al., 2017; Ramakrishna et al., 2018). However, in current crop research studies, InDel variant mining and marker development is usually focused on a single type of dimorphic InDel, which does not reflect the overall InDel diversity across the genome (Zhao et al., 2017; Wang et al., 2018; Paudel et al., 2019; Wang et al., 2020; Fei et al., 2021; Hechanova et al., 2021; Liu et al., 2022; Seki, 2022). Only small, dimorphic InDels have been extensively examined in whole plant genomes; InDels of various lengths and types, which have important value and application potential in genetic research, have not been fully elucidated and systematically analyzed.

Despite maize, rice, and soybean being significant model plants in fields of genetic breeding, gene function, evolutionary genetics, and plant diversity research (Carter et al., 2004; Hirsch et al., 2014; Li et al., 2014; Schatz et al., 2014; Wing et al., 2018; Li et al., 2020; Liu et al., 2020; Hufford et al., 2021; Qin et al., 2021), several factors have inhibited the comprehensive development of InDel markers within these species. First, mining InDel loci of different lengths has traditionally relied on the examination of a single reference genome, which leads to genomic bias and the inevitable loss of some InDel loci. Furthermore, these mining strategies based on single reference genomes often use second-generation short reads for mapping, but many important InDels associated with agronomic traits are too large to be easily detected with Illumina short reads (Liu et al., 2020; Matar and Melzer, 2021). Therefore, the identification of medium (3–50 bp) and large (≥50 bp) InDels requires the use of a multi-genome collinearity approach rather than a single-genome mining strategy. Second, almost all research related to InDel mining and marker development has focused on dimorphic InDels, but an InDel itself is polymorphic (Li et al., 2019; Bennett et al., 2020). Third, Multi-InDel analysis, which examines the number of useful InDel polymorphisms while retaining the advantages of SNPs and SSRs, has been applied in medicinal research but not yet in plants (Sun et al., 2019; Qu et al., 2020). Fourth, Perfect InDels, which exist naturally in both animals and plants, are single-copy, mainly dimorphic, easily detected and typed, and characterized by a high minor allele frequency (MAF), conserved flanks, and a length of 3–10 bp; thus, they are compatible with multiple detection platforms. Perfect InDels can be used to track certain key traits and identify cultivars but have not been mined in model crop systems. Furthermore, no studies have been performed to identify and analyze InDel loci with insertions or deletions in the form of repeating units or to uncover InDel loci embedded in other types of variants. In summary, the types, and lengths of InDel loci in crop plants have not been systematically obtained and sorted. Overcoming the above-mentioned problems associated with InDel variation mining and application to model crops such as maize, rice, and soybean would provide an important foundation for breeding and domestication research on other crop species.

To comprehensively capture and analyze the intricacies of InDel variations in crops, our initial step involved formulating a strategy based on Omni InDel. In this approach, variation mining in the studied crop species was carried out using multiple assembled genomes within the species; simultaneously, all collected InDel variants within the genome were integrated into a thorough variation map of InDels comprising dimorphic, polymorphic InDels; Perfect InDels; and Multi-InDels that range from 1 bp to several Mb. We also classified types of SSRs and non-SSR InDels. To enrich our InDel variation map and provide plant researchers with richer variation information, we then collected information on variable InDel loci in maize, soybean, and rice and constructed Omni InDel databases for these species. We also established a multi-species Omni InDel database website for data sharing and dissemination. Finally, we selected several loci for chip verification and, using maize as an example, identified 3,587 germplasm resources to verify the effectiveness of the developed markers.

## Materials and methods

### Small and medium Omni InDel identification

To identify small Omni InDels (SOIs, 1~2bp) and medium Omni InDels (MOIs, 3~50bp), the illumina paired-end reads of each maize species were mapped to genome assembly B73 with BWA (v. 0.7.17-r1198-dirty) (Li, 2013) with default settings. The bam files obtained with BWA were sorted by SAMtools (v. 1.9) (Danecek et al., 2021). The variants were called using GATK (v. 4.2) (McKenna et al., 2010) with default settings, and the InDels were extracted on the basis of the GATK results.

### Large and huge Omni InDel identification with MUMMER

To identify large Omni InDels (LOIs, 50–1000 bp) and huge Omni InDels (HOIs, >1000 bp), we aligned the 11 maize inbred line assemblies to the B73 reference genome based on Nucmer (v. 4.0.0beta2) (Marçais et al., 2018) with the parameters “—mum -g 1000 -c 90 -l 40.” Then, the alignment files were filtered to generate 1-to-1 mapping by delta-filter with the parameters “-m -i 90 -l 100.” The Nucmer output was analyzed using SyRI (https://github.com/schneebergerlab/syri) (Goel et al., 2019) with default parameters to identify variation. According to the sequence variation definitions in SyRI outputs, we extracted the InDel variation from the raw results.

### Germplasm genetic pattern analysis

A phylogenetic tree was constructed using medium Omni InDels (MOIs) by unweighted pair group method with arithmetic mean (UPGMA) in the R (v. 3.5.1) package “phangorn” (Schliep, 2011). The data used to construct the tree were from a genetic distance matrix calculated by the “poppr” (Kamvar et al., 2014) package in R.

Principal components analysis (PCA) of InDel loci from 3,558 *Zea mays* inbred lines was also conducted in PLINK v1.90b6.21 64-bit (http://pngu.mgh.harvard.edu/purcell/plink/) (Purcell et al., 2007) with the same dataset. The PCA results further confirmed and supported the clusters classified by the phylogenetic tree.

### Development of the Omni InDel web-based database

The Omni InDel online platform was built using the Next.js framework. Next.js is a lightweight React.js server-side rendering application framework that connects web pages to application programming interfaces through a multifunctional router. This platform integrates powerful JavaScript tools and makes it easier to build and deploy the released versions through the Webpack package. We standardized the code format using the Prettier tool, used the Eslint tool to eliminate obvious errors from the code, and used Babel to maintain compatibility between normal JS and ES6. On the front end, we used Ant Design, which is a front-end user interface component based on the React.js.

## Results

### Omni InDel statistics of crops

Omni InDel sites encompass insertion-deletion variations that range from 1 bp to 9.4 Mb. Specifically, InDel sites spanning 1 bp to 2 bp are classified as small Omni InDel (SOIs), those with lengths from 3 bp to 50 bp were considered MOIs, those with lengths from 50 bp to 1000 bp were considered LOIs, and those with lengths >1000 bp were considered HOIs.

**Figure 1:**
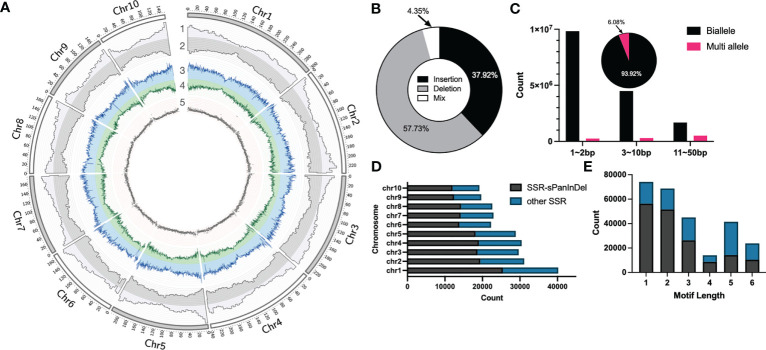
Statistical overview of small Omni InDels (SOIs) and medium Omni InDels (MOIs). **(A)** Circos (Krzywinski et al., 2009) display of SOI and MOI density distribution. 1, Gene density of B73. 2, Distributions of SOI and MOI. 3, Deletion density. 4, Insertion density. 5, Distribution of SSR-InDels in chromosomes, the window size for density calculation is 5 million bp. **(B)** The proportion of insertion, deletion and insertion-deletion mixed type sites in the MOI and SOI sets. **(C)** Counts of biallele and multi-allele InDels of different sizes (1–2 bp, 3–10 bp, 11–50 bp), and the pie is the ratio of biallele and multi-allele InDels. **(D)** Numbers of SSRs in each maize chromosome and the ratio between Omni InDel SSRs and other SSRs. **(E)** Numbers of SSRs with different motif lengths and the ratio between Omni InDel SSRs and other SSRs.

In order to construct Omni InDel datasets for SOI and MOI sites less than 50 bp in length, next-generation paired-end sequencing (average 4×) on 438 maize inbred lines commonly used in breeding at home and abroad was performed, followed by InDel calling using GATK with B73 as the reference genome. After filtering and screening, 17,820,869 InDels were obtained. These sites were predominantly distributed in the chromosome arms and less so in the centromeric region (**Figure 1A**). Comparison with the annotated gene positions of the B73 genome revealed that 15.49% of the sites were distributed in the gene region, and the others were distributed in the intergenic region. Insertions and deletions accounted for 37.92% and 57.73% of the sites, respectively. In addition, 4.35% of the sites showed characteristics of both insertions and deletions (**Figure 1B**). These InDel sites could not be traditionally classified; therefore, these InDel named as Mixed-Omni InDel. Among the non-mixed sites, more than 60% were within 1–2 bp; additionally, 6.08% had more than two alleles, and the rest were biallelic sites (**Figure 1C**).

The polymorphisms of SSR molecular markers manifest as different numbers of motifs in different plant varieties, and sequence scanning and identification of motifs of an assembled genome is generally used when mining such sites. Utilizing Misa, a total of 267,041 SSR sites was identified within the B73 reference genome (motif repeat numbers: 1-10, 2-6, 3-5, 4-4, 5-3, and 6-3), of which a minimal number were SSR sites with a motif length of 4. It was found that a total of 167,590 SSR sites existed in the form of motifs in the sites corresponding to Omni InDels, which accounted for 62.76% of all SSR sites, when the location and motif type of the SSR sites in SOIs and MOIs (but not LOIs and HOIs) were compared. It was found that some of these Omni InDel sites with two or more alleles exhibited tandem repeat motifs with different numbers of repetitions; they presented as double polymorphisms of InDel and SSR sites, which we propose could be used as Omni InDel sites with different allele types (**Figures 1D, E**). Therefore, sites with both SSR and InDel characteristics were referred to as SSR-Omni InDel sites.

**Figure 2:**
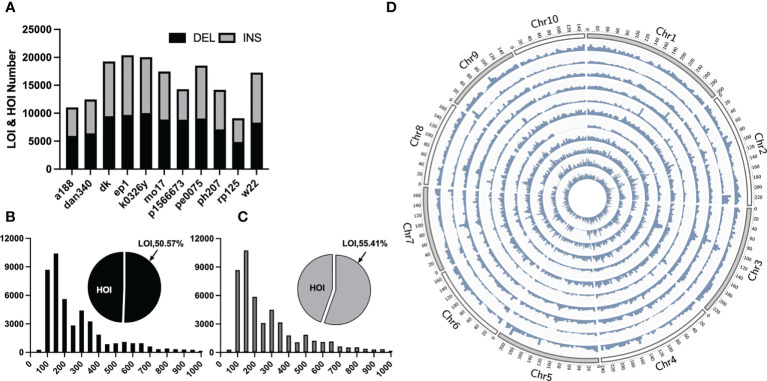
Statistical overview of large Omni InDels (LOIs) and huge Omni InDels (HOIs). **(A)** Numbers of LOIs and HOIs of 11 maize inbred lines and the gray and black blocks represent the proportions of DEL and INS respectively within LOIs and HOIs. **(B)** Proportion and distribution of different lengths of the deletion-type LOIs. **(C)** Proportion and distribution of different length of the insertion-type LOIs. **(D)** Density distribution of LOIs and HOIs in 11 maize inbred lines; from the outer ring to the inner ring: A188, Dan340, DK, EP1, K0326Y, Mo17, P1566673, PE0075, PH207, RP125, W22, the window size for density calculation is 5 million bp.

Traditional mutation mining based on next-generation sequencing technology can only obtain small InDels, whereas pan-genomic analysis has been recently become popular, in which large structural variants, such as presence/absence variations (large and huge InDels) can be obtained by collinearity between genomes. For LOI and HOI sites with the lengths of ≥50 bp, collinear comparison was performed on 11 portions of maize genomes assembled with third-generation sequencing data and the B73 reference genome and a total of 92,196 LOI sites and 81,898 HOI sites in the 11 maize inbred lines were obtained (**Figures 2B, C**). Most of these sites were evenly distributed on chromosomes, although some sites were concentrated in the chromosome arms, and a few were distributed in the centromere region (**Figure 2D**). The greatest number of sites was present in the EP1 variety (LOIs and HOIs: 20,387), and the smallest number of sites was present in the RP125 variety (9,103). Insertions and deletions accounted for 49.26% and 50.74% of InDels, respectively (**Figure 2A**). Among the 11 maize inbred lines, the proportion of deletions to insertions was between 0.91 and 1.6, with P1566673 accounting for the largest proportion (deletions: 8,819/insertions: 5,479).

Omni InDels are variants that exist in a variety of species, but their markers have not been completely developed. With the continuous improvement of third-generation sequencing technology and bioinformatics tools, complete Omni InDel markers for a variety of organisms have been developed to meet the needs of complex analyses in plant sciences, crop breeding, forensic science, human genetics, evolutionary science, and animal breeding-related fields. To fully elucidate the role of Omni InDels in different crops and enrich the site pool of Omni InDels, InDels from rice (Yan et al., 2020; Shang et al., 2022) and soybean (Liu et al., 2020) were collected in addition to maize and built an Omni InDel database for each species. (Http: https://omni-indel.plantdna.site)

### Development and validation of perfect InDels and germplasm analysis

Mutation mining is very common in crop research, but developing mutation sites into usable molecular markers requires mining and experimental verification. To establish the reliability and applicability of our developed Omni InDel database, especially with respect to molecular marker genotyping and further analysis, we proceeded to filter the MOIs and constructed a Perfect InDel set composed of single-copy, mainly dimorphic, easily genotyped InDels that are characterized by a high MAF (≥0.05) and a mutation length of 3–10 bp. Thus, the Perfect InDels were compatible with multiple detection platforms, such as polyacryamide gel electrophoresis (PAGE), fluorescent capillary electrophoresis (CE), Kompetitive Allele-Specific PCR (KASP) and sequencing. We first extracted the upstream and downstream 30-bp sequences of the sites with the above characteristics, performed BLAST comparison in the genome, and screened out 4,246,656 sites with at least one flank being a single copy upstream or downstream; then, the sequences were filtered under the condition of MAF ≥0.05. Finally, 1,212,345 sites were obtained, and these sites formed the set of Perfect InDels.

To validate the maize Perfect InDel sites, 4,538 InDels that covered 10 maize chromosomes in the Perfect InDel set were arbitrarily selected. The selected InDels varied in length from 3 bp to 10 bp; 3,558 maize inbred lines at home and abroad were collected and genotyped using the InDels selected above using the chip platform. The results demonstrated that the selected Omni InDel sites facilitated satisfactory genotyping across all maize inbred lines, and the average number of missing sites per inbred line was 87, the smallest number of missing sites of inbred line samples was only 7, and the average missing rate of samples was 1.93%.

**Figure 3:**
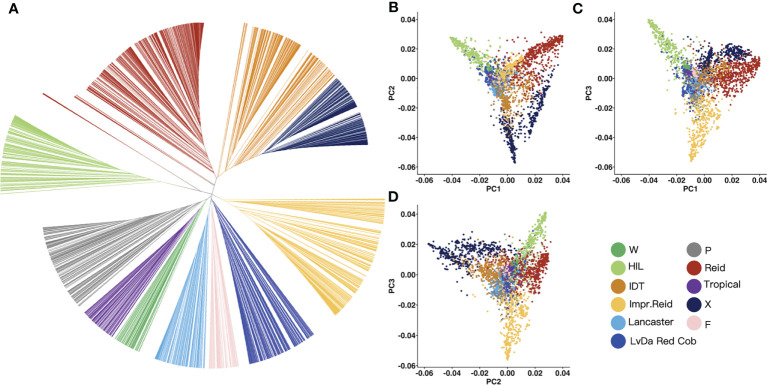
Phylogenetic tree and PCA result for 3,558 maize inbred lines. **(A)** UPGMA tree constructed based on the genetic distance matrix between maize inbred lines, 3,558 samples were genetically clustered into the following 11 groups: tropical, flint inbred lines (F), waxy (W), LvDa Red Cob, HZS-improved line (HIL), Improved Reid (Impr.Reid), Reid, Lancaster, P(P78599 line), Iodent (IDT), and X. **(B–D)** Scatter plots of the first principal component (PC1) versus PC2 and PC1 versus PC3 and PC2 versus PC3.

In order to classify the germplasm groups among these 3,558 maize inbred lines, phylogenetic tree was built (**Figure 3A**) for these lines based on the genetic distance between lines constructed with the selected InDels mentioned above using maximum likelihood. Based on the genetic distance between the samples, the 3,558 maize inbred lines were divided into 11 groups. Of these 11 groups, the Reid group had the most inbred lines (676), followed by improved Reid (527), and the total proportion of the two groups reached 33.7%. Among them, some maize inbred lines in the HZS-improved line group had similar genetic distances, which may be related to the frequent improvement of some elite inbred lines in the HZS-improved line group by breeders, resulting in more HZS-improved line group with very similar genetic backgrounds.

Then, PCA was performed to test the reliability of the phylogenetic results. According to the PCA results, PC1, PC2, and PC3 represented 21.84%, 14.11%, and 12.66% of the total variation, respectively (**Figures 3B–D**). There was a long genetic distance between the HZS-improved line group, Reid group, and X group. The genetic distance between the Reid group and the improved Reid group was small because the improved Reid group was developed from the Reid group.

### Development of multi-InDels with Omni InDels

The concept of Multi-InDels was first introduced in the field of human research by Qu et al., 2020. Based on this concept, to expand the application of Omni InDels in crops, a set of Multi-InDels was developed that can be applied in crop research based on Omni InDels that met the following conditions: (1) mutation length between 3–10 bp, namely select sites in the MOI set; the missing rate is <50% and the MAF is >0.1; (2) each Multi-InDel contains 3 or more MOI sites; (3) the combination of variation length of InDels in the Multi-InDel namely the length of each possible theoretical haplotype of Multi-InDel should be different to facilitate detection of each possible allele on the detection platform; (4) each site in the Multi-InDel should be dimorphic; (5) to ensure that PCR can be conducted and that the sites are compatible with fluorescent capillary electrophoresis platforms as well as sequencing platforms, the maximum length of each Multi-InDel is within 200 bp; (6) the distance between two adjacent Multi-InDel sites is more than 200 bp.

Given these six core parameters, Multi-InDels offer the advantages of high polymorphism and can be detected using both sequencing and fluorescence platforms, thus presenting significant practical value.

A total of 441,572 candidate sites, each with a MAF of at least 0.1, were pinpointed when we examined the maize MOI locus database. Next, using the sliding window approach, 22,774 candidate Multi-InDels were selected, which included a total of 81,976 MOIs. The maximum distance between MOIs within the candidate Multi-InDels did not exceed 200 bp.

**Figure 4:**
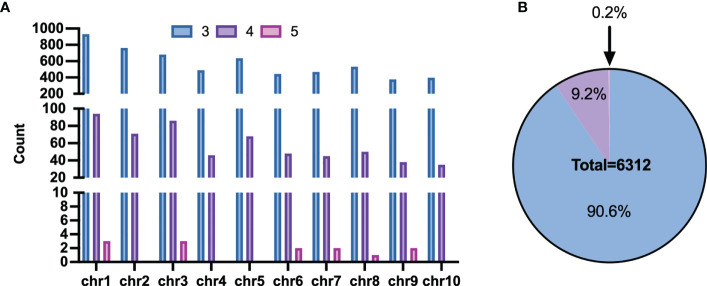
Distribution of Multi-InDels. **(A)** Count of Multi-InDels containing 3-5 InDels in each chromosome. **(B)** Proportion of Multi InDels containing 3–5 InDels.

However, some haplotypes of these candidates had the same PCR length, which made genotyping impossible. The length of haploid genotypes that may appear in the candidate Multi-InDels was filtered to address this. Finally, 6,432 sites were obtained with different lengths for all theoretical haplotypes. 20 sites with two adjacent Multi-InDels spaced within 200 bp of each other was excluded, and eventually revealed 6,312 Multi-InDel sites (**Figure 4A**). These sites were distributed across chromosomes, and they contained up to 5 MOIs at most which accounting for the proportion 0.2% and 3 MOIs at least which accounting for 90.6% (**Figure 4B**).

### Establishment of an Omni InDel network database

**Figure 5:**
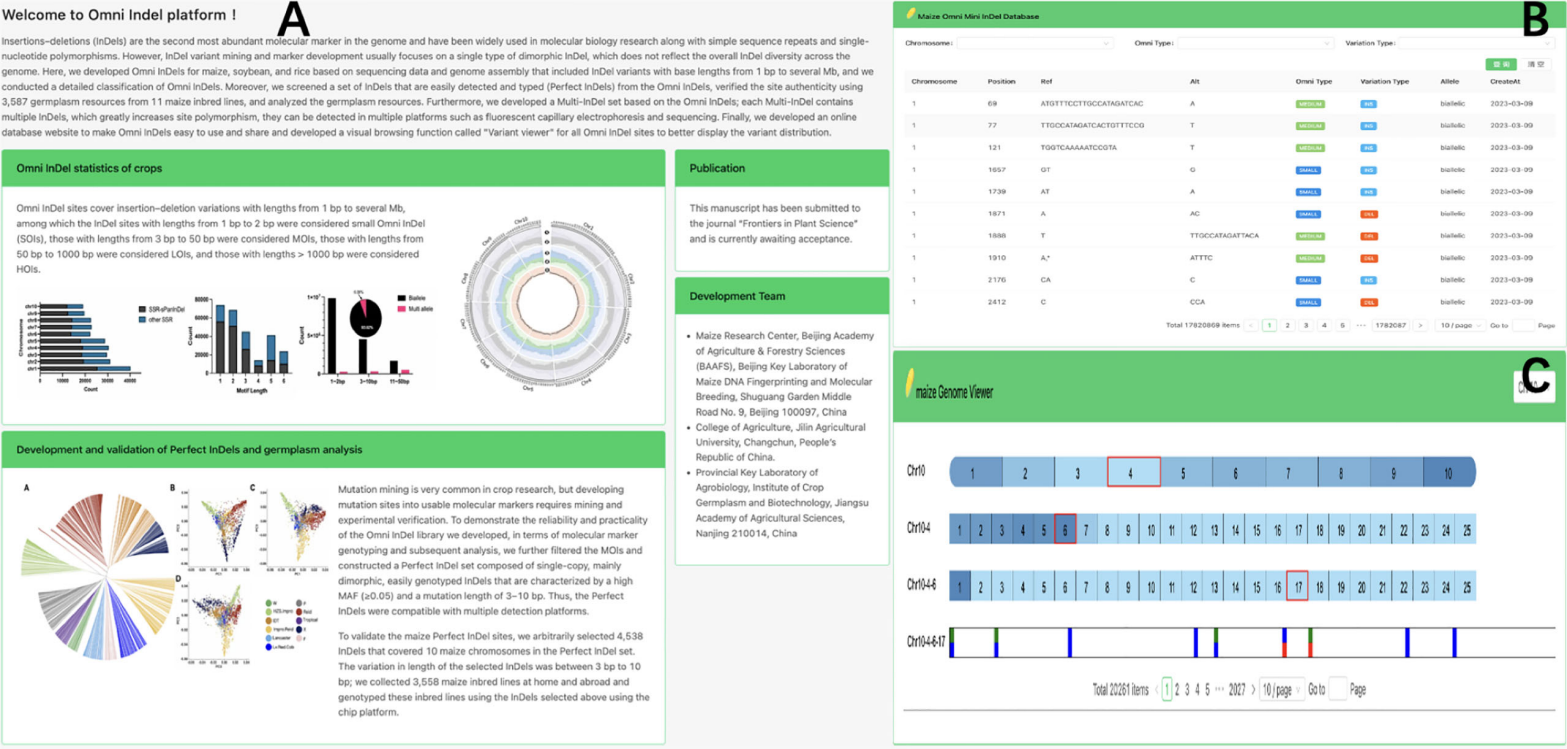
Display Omni InDel network database. **(A)** The homepage of Omni InDel website **(B)** Data browser of Omni InDel. **(C)** Screenshot of the”Variant Viewer”.

To enhance the accessibility of Omni InDel resources, we developed a web-based database (https://omni-indel.plantdna.site) that contains all Omni InDel information for maize, rice, and soybean (**Figure 5**). Researchers can effortlessly access, browse, and download information on variable loci of interest from the Omni InDel database.

The Omni InDel online platform provides two core functional modules: “Browse” and “Variant viewer.” Among these, the “Browse” module, which utilizes MySQL for database storage, provides key information including “location details,” “Omni classification,” and “mutation specifics” of all types of Omni InDels; this facilitates seamless switching between maize, rice, and soybean. Additionally, all the data can be downloaded. The “Variant viewer” function is a variation display of newly developed online interactive genome structure. The HTML5 Canvas function was used to draw the distribution map of variants on chromosomes and added interactive functions such as scrolling and clicking. By clicking on different blocks, users can shift the visualization area, resulting in a clear representation of chromosomal variations. As a browser feature, Canvas allows for superior customization and browser rendering efficiency in genome visualization compared to other tools.

Using maize as an example, the web database also facilitates variation visualization as a part of its browsing functionality, which allows researchers to browse the whole genome density distribution of variation at different scales and easily choose a single variation from the millions recorded. Four different types of Omni InDel are displayed in the window and become interactive points. After the users click, detailed variation information of each site is displayed.

## Discussion

This study presents the Omni InDel database for multiple crops (maize, soybean, and rice). It offers a rich reservoir of insertion and deletion molecular variation information to support research related to molecular-assisted breeding, population structure genetic analysis, and differentiation of breeding varieties. The Omni InDel database can serve as a critical resource for researchers working on the breeding of new crop cultivars. This database contains InDels for which the length varies from 1 bp to several Mbp, which could allow researchers to flexibly choose the molecular variation that meets their needs. Moreover, InDel has an incomparable advantage over SNP in terms of the length of the variation type. Firstly, in the detection of molecular markers, SNP is mainly detected by methods such as sequencing, KASP, etc., while InDel can be detected by simple gel electrophoresis. Secondly, InDel may cause more severe mutations than SNP, because some relatively large InDels may even cause chromosomal structural changes.

More than 60% of SSR loci were found in the Omni InDel database. These are dual-characteristic variable loci, and some of them showed polymorphism in the database. Hence, when mining SSR molecular markers, it’s crucial to filter the assembled genome by motif and employ the locus information in the Omni InDel database for dual verification of the mined SSR loci. This process aids in the identification of dual-characteristic variable loci. This can effectively reveal many reliable SSR loci, and we can also eliminate InDel loci with SSR characteristics when necessary.

To extend the application scope of Omni InDels, we developed Multi-InDels based on Omni InDels. The polymorphism of Multi-InDels that contained 3–5 InDels within 200 bp greatly increased compared with that of single InDels, with 8 theoretical haplotypes for Multi-InDels containing 3 InDels and 32 theoretical haplotypes for Multi-InDel containing 5 InDels. The polymorphism of Multi-InDels is not less than that of SSRs. Moreover, because SSR-InDels were removed from Multi-InDels, the chance of stutter in the amplification process of this locus was greatly reduced. Moreover, DNA fingerprinting reveals that SSRs exhibit polymorphism through the insertion or deletion of several base motifs. The size difference in the amplification fragment between different SSR haplotypes corresponds to a multiple of the number of consecutive motifs, thereby adding complexity to the fingerprinting process. All possible amplification lengths of Multi-InDels are known and the amplification products will not show stutter, which makes fingerprinting simpler and clearer. This is the first Multi-InDel database on crops since the release of Multi-InDel database in human research in 2020. This database should be verified and applied in crop-related research.

In addition to the development of this Omni InDel database for multiple crops using maize as an example, we verified some MOIs that were arbitrarily chosen from the whole database to genotype 3,558 domestically and foreign-collected maize inbred lines on an array-based platform. Then, we carried out germplasm analysis using these genotype fingerprints, and the results supported Omni InDels as an effective resource for germplasm analysis. Furthermore, those markers developed from the database (such as Perfect InDels) can also be compatible with multiple detection platforms include array based and KASP which are high throughput platform. These characteristics make them high throughput, high compatible marker (HCM).

A web-based Omni InDel database was developed to facilitate the sharing and dissemination of Omni InDel data, making it readily accessible for browsing on this website.

## Data availability statement

The datasets presented in this study can be found in onlinerepositories. The names of the repository/repositories and accessionnumber(s) can be found below: NCBI BioProject accessionnumber: PRJNA976755.

## Author contributions

FW and HZ designed the experiments and managed the project. JZ managed the project. ZL performed the data analysis and wrote the manuscript. YKZ and WY reviewed and edited the manuscript. YLZ performed the data curation and validation. LX and LZ provided the resource data. All authors contributed to the article and approved the submitted version.
